# Effects of Grammaticality and Morphological Complexity on the P600 Event-Related Potential Component

**DOI:** 10.1371/journal.pone.0140850

**Published:** 2015-10-21

**Authors:** Alison S. Mehravari, Darren Tanner, Emma K. Wampler, Geoffrey D. Valentine, Lee Osterhout

**Affiliations:** 1 Program in Neuroscience, University of Washington, Seattle, Washington, United States of America; 2 Department of Linguistics, Beckman Institute for Advanced Science and Technology, Neuroscience Program, University of Illinois at Urbana-Champaign, Champaign-Urbana, Illinois, United States of America; 3 Department of Psychology, University of Washington, Seattle, Washington, United States of America; The National Institutes of Health, UNITED STATES

## Abstract

We investigated interactions between morphological complexity and grammaticality on electrophysiological markers of grammatical processing during reading. Our goal was to determine whether morphological complexity and stimulus grammaticality have independent or additive effects on the P600 event-related potential component. Participants read sentences that were either well-formed or grammatically ill-formed, in which the critical word was either morphologically simple or complex. Results revealed no effects of complexity for well-formed stimuli, but the P600 amplitude was significantly larger for morphologically complex ungrammatical stimuli than for morphologically simple ungrammatical stimuli. These findings suggest that some previous work may have inadequately characterized factors related to reanalysis during morphosyntactic processing. Our results show that morphological complexity by itself does not elicit P600 effects. However, in ungrammatical circumstances, overt morphology provides a more robust and reliable cue to morphosyntactic relationships than null affixation.

## Introduction

Prior research has identified several event-related brain potentials (ERPs) that are robustly sensitive to aspects of real-time language comprehension. One of these potentials, the P600 effect, is a positive-going wave that onsets at about 500 ms after presentation of the anomalous word and persists for several hundred ms. P600 effects are routinely elicited by anomalies involving grammatical agreement, tense, case, and verb subcategorization [1–9]. By contrast, the N400 component is sensitive to properties of words (such as lexicality and word frequency), a word’s predictability, and the “semantic fit” between a word and its context (see [10]). These ERP responses have been used to study a wide array of topics pertaining to real-time language comprehension, including the processing of dissociation between sentence structure and meaning (e.g., [3,11], cf. [12,13]) and neurocognitive changes that accompany increasing proficiency in a second-language [14–16].

However, fundamental questions remain about the range of stimulus and task properties that elicit and modulate these language-sensitive ERP effects. Here, we focus on a possible confounding between two stimulus variables, namely, ungrammaticality and morphological complexity. The stimuli from Osterhout and Nicol [3], a frequently-cited paper, make this issue clear. In that study, ERPs elicited by critical words in grammatically correct sentences like “*The cat will* *eat* *the food”* (critical word underlined) were compared to ERPs elicited by critical words in grammatically incorrect sentences like “The cat will * *eating* *the food”*. “Eating” is both grammatically incorrect and morphologically more complex than “eat”; “eating” contains the extra morpheme “-ing”. The Osterhout and Nicol paper is only one of many in which morphological complexity and grammaticality have been conflated (see [17] for discussion and review). Note that the confound in Osterhout and Nicol’s study and in similar studies is not a fatal flaw, in that the logic of the interpretations were valid regardless of whether such a confounding was present. Nonetheless, the confound does make it difficult to identify the exact cause of the difference in brain responses between conditions, i.e., whether the P600 resulted solely from the effects of ungrammaticality, or a combination of ungrammaticality plus complexity.

A possible remedy to this confound is to hold the morphological form of the critical word constant, while varying the pre-critical context (e.g., *The cat was* *eating* *…*versus The cat will * *eating* *…*). However, this remedy immediately runs into two problems. First, the pre-critical word varies across conditions. This can have important consequences for interpretation of ERPs time-locked to the critical word, because ERPs elicited by the pre-critical word can spill over and contaminate the signal elicited by the word of interest. This is especially a problem in designs where the pre-critical word differs across conditions in length or word category (see [17,18] for critical discussions of this issue). Second, these sorts of contextual manipulations can lead to predictability of a sentence’s grammaticality *before* the critical word is encountered. In the example provided above, assuming that other sentences in this grammatical/ungrammatical comparison were constructed similarly, the auxiliary verb *will* is fully predictive of the upcoming verb’s grammaticality. That is, over the course of the experiment, participants would be able to develop expectations about upcoming words’ grammaticality before they are actually encountered. Including a sufficient number of filler stimuli that mask the predictability of ungrammatical forms is one remedy to this issue. However, given the large number of stimuli needed for ERP research, this can quickly lead to experimental paradigms that are unduly long and taxing for participants.

One solution to these problems is to use balanced (factorial) designs that are fully crossed in terms of grammaticality and morphological complexity (e.g., have four conditions in which the simple and complex critical words are each used in both grammatical and ungrammatical sentences). In analyzing data from balanced designs, one would simply average across levels of complexity at the target word, and then test for the main effect of grammaticality. In many cases balanced designs are appropriate and adequate to overcome the problems associated with baseline confounds for context manipulations and complexity/predictability confounds for target manipulations (see [19] for an example). However, even in cases where ungrammatical stimuli at both levels of complexity elicit P600 effects, there may be theoretical reasons to investigate interactions between grammaticality and complexity. For example, affixation itself might have an influence on the brain response to words as they are encountered during reading, and the brain response to ungrammatical features might be influenced by whether or not the feature is expressed through overt inflectional morphology.

Numerous electrophysiological and electromagnetic studies have indicated that readers decompose a morphologically complex word into its component morphemes (e.g., [20–28]), though these studies typically used either single-word or priming paradigms, where decomposition is indexed by modulations of the N400 or other earlier components. Grammatical complexity at the sentence level typically impacts the P600 component, even in the absence of any type of anomaly [5,29]. What is unclear from this work is whether morphologically complex sentence-embedded words may also elicit a P600-like effect in the same way as words in syntactically complex sentences. That is, decomposition processes engaged during sentence reading may impact brain responses, leading to at least partially additive effects of grammaticality and complexity on the P600.

On the other hand, some sentence processing models predict that grammaticality will interact with morphological complexity. Such interactions are predicted by models that posit both *active prediction* of grammatical affixes, and *memory searches* for the “controller” of verb tense (the entity in the sentence that determines verb tense; [6]) once the affixed verb has been encountered in the sentence. It is now well established that comprehenders make active predictions about upcoming linguistic material at multiple levels, including word forms, semantics, morphology, and syntax [30–41]. For example, if a sentence contains a verb in the past progressive tense (e.g. *The sheep were* …), the reader could predict a subsequent verb marked with the–*ing* suffix (e.g., *grazing*). Conversely, if the sentence contained a modal verb (e.g., *The sheep should …*), the reader could predict an upcoming verb without overt inflection (e.g., *graze*). In cases where these predictions are met, no processing difficulty is predicted, and brain responses should not vary based on the morphological complexity of the target word. The newly encountered word can easily be integrated into the predicted linguistic representation, leading to a lack of P600 effects (see also [42]). However, in cases where bottom-up detected words mismatch with features generated by the prediction, the reader searches through his or her representation of the sentence in working memory to find the entity acting as the grammatical “controller” of verb morphology (in this case, the verb *were*) [8,43–52].

From this perspective, the ease of sentence comprehension varies as a function of how well the successive words in the sentence allow accurate predictions about upcoming words, and also the quality of cues provided by words triggering retrievals and the degree of overlap with potential targets. (see [43] for an explicit, computationally-implemented model of this). This prediction/memory search account therefore predicts interactions between grammaticality and complexity that might be manifested in P600 amplitude. For the ungrammatical sentence “*The sheep were graze*…”, the context should lead to a prediction that the verb will be in the present participle form (*grazing*). The absence of the expected grammatical affix should elicit a P600 effect. However, because the verb appeared in its base form with no inflectional morphology, there may be no subsequent search through working memory to identify the “controller” of the verb form (*were*; i.e., a lack of a search due to a lack of a triggering morpheme), or because the form *graze* is a base verb form lacking for features, it will not clash in features with any potential controllers held in working memory. By contrast, for the ungrammatical sentence “*The sheep should grazing…”*, the modal form of the verb leads to the prediction of the uninflected base verb form (*graze*). However, the presence of the unexpected grammatical affix *-ing* will also initiate the search through working memory for a controller of agreement (a verb in progressive tense). Because *grazing* is specified for tense and aspectual features, no such controlling verb can be found in the sentence. This sentence is therefore “anomalous” in two ways: by virtue of the ungrammaticality, and by virtue of an unsuccessful working-memory search for an appropriate licensing verb. This model therefore predicts an interaction between grammaticality and the morphological complexity, such that the P600 effect to critical words in the anomalous sentences will be larger for the inflected form of the anomalous verb.

## Method

### Participants

Participants were 14 native English speaking college students. All participants were all strongly right-handed, as assessed by an abridged version of the Edinburgh Handedness Inventory [53] and had normal or corrected-to-normal vision. One participant reported having become fluent in German at age 16; all other participants were only fluent in English. All participants were of age 18 or over. Specific age and gender information was not collected from 1 of the 14 subjects; of the 13 subjects from whom this information was collected, 4 were female (self-reported gender), and the average age was 19.6 years (range: 18–25 years). Participants provided written informed consent and received course extra credit for taking part in the study. All experimental procedures were approved by the University of Washington Institutional Review Board.

### Materials

Stimuli were 120 sentence quadruplets in a fully crossed 2 (morphological complexity) by 2 (grammaticality) design. Sentences were either grammatically correct or contained a violation of constraints on tense morphology, and the critical word was either morphologically simple or morphologically complex, resulting in four versions of each sentence (see Table 1 for an example). Critical words were verbs in either the base form (e.g., graze) or the present participle form (e.g., grazing). 120 different verbs were used, and the verbs were chosen so that the average written word-form log frequency (provided in the CELEX2 database [54]) of the base form and present participle form of all 120 verbs was not significantly different (base form average frequency = 1.233, present participle form average frequency = 1.230, *t* = 0.067, *p* = 0.946). The word immediately preceding the critical word was either *was/were* or a modal verb (*should/could/would/will/can/must/may*). The four versions of each sentence were distributed across four experimental lists, such that each participant only saw one version of each sentence, and there were 30 sentences per condition in each list. Each list contained an additional 60 filler sentences, all of which were grammatically correct. In total, each list contained 180 sentences. The sentence order in each list was randomized, and lists were divided into 3 blocks of 60 sentences each. S1 Table lists all experimental and filler sentences.

**Table 1:** Example experimental stimuli.

Grammaticality	Morphological Complexity	Sentence
Grammatical	Simple	*The sheep should* *graze* *in the pasture*.
Ungrammatical	Simple	*The sheep were* *graze* *in the pasture*.
Grammatical	Complex	*The sheep were* *grazing* *in the pasture*.
Ungrammatical	Complex	*The sheep should* *grazing* *in the pasture*.

Note: The critical word for ERP averaging is underlined.

### Procedure

Participants were tested in a single session lasting no more than 120 minutes. Each participant was randomly assigned to one of the stimulus lists. During testing, participants were seated in a comfortable recliner in front of a CRT monitor. Participants were instructed to relax and minimize movements and eye blinks while silently reading each sentence. Each trial consisted of the following events: a blank screen for 1000 ms, followed by a fixation cross, followed by a stimulus sentence presented one word at a time. The fixation cross appeared on the screen for 500 ms followed by a 400 ms ISI. Each word of the sentence appeared on the screen for 300 ms followed by a 350 ms ISI. After the final word of the sentence, there was a 1000 ms blank screen, followed by a “yes/no” prompt. Participants were instructed to give a sentence acceptability judgment at the “yes/no” prompt, where “yes” was the response for sentences that were correct in all ways and “no” was the response for sentences that contained any sort of error. The “yes/no” prompt remained on the screen until participants responded “yes” or “no”; as soon as a response was given, presentation of the next sentence began. Participants were randomly assigned to use either their left or right hand for the “yes” response.

### Data acquisition and analysis

Continuous EEG was recorded from 19 tin electrodes attached to an elastic cap (Electro-cap International) in accordance with the 10–20 system [55]. Eye movements and blinks were monitored by two electrodes, one placed beneath the left eye and one placed to the right of the right eye. Electrodes were referenced to an electrode placed over the left mastoid. EEG was also recorded from an electrode placed on the right mastoid to determine if there were differential experimental effects detectable on the mastoids. No such effects were found. EEG signals were amplified with a bandpass of 0.01–40 Hz (-3db cutoff) by an SAI bioamplifier system. ERP waveforms were filtered offline below 30 Hz. Impedances at scalp and mastoid electrodes were held below 5 kΩ and below 15 kΩ at eye electrodes.

Continuous analog-to-digital conversion of the EEG and stimulus trigger codes was performed at a sampling frequency of 200 Hz. ERPs, time-locked to the onset of the critical word in each sentence (underlined in the examples in Table 1), were averaged offline for each participant at each electrode site in each condition. Trials characterized by eye blinks, excessive muscle artifact, or amplifier blocking were not included in the averages. 3.70% of all trials were rejected, and the rejection rate did not differ significantly between experimental conditions: *F*(3,52) = 0.024, *p* = 0.995). ERPs were quantified as mean amplitude within a given time window. All artifact-free trials were included in the ERP analyses. In accordance with previous literature and visual inspection of the data, the following time windows were chosen: 300–500ms (LAN/N400), and 500–900ms (P600), relative to a 100ms poststimulus baseline. A poststimulus baseline was used because there were moderate differences in the prestimulus baseline, likely a result of needing to use different pre-critical words across conditions.

Differences between conditions were analyzed using a repeated-measure ANOVA with two levels of morphological complexity (simple, complex) and two levels of grammaticality (grammatical, ungrammatical). Data from midline (Fz, Cz, Pz), medial (right hemisphere: Fp2, F4, C4, P4, O2; left hemisphere: Fp1, F3, C3, P3, O1), and lateral (right hemisphere: F8, T8, P8; left hemisphere: F7, T7, P7) electrode sites were treated separately in order to identify topographic and hemispheric differences. ANOVAs on midline electrodes included electrode as an additional within-subjects factor (three levels), ANOVAs on medial electrodes included hemisphere (two levels) and electrode pair (five levels) as additional within-subjects factors, and ANOVAs over lateral electrodes included hemisphere (two levels) and electrode pair (three levels) as additional within-subjects factors. The Greenhouse-Geisser correction for inhomogeneity of variance was applied to all repeated measures on ERP data with greater than one degree of freedom in the numerator. In such cases, the corrected *p*-value is reported.

## Results

Results from the end-of-sentence judgment task showed that participants were highly accurate in judging the acceptability of the sentences (grammatical mean proportion correct = .92, SE = .02; ungrammatical mean proportion correct = .96, SE = .01). Accuracy in judging the simple versus complex sentences (Table 2) was not significantly different (grammatical simple v. complex stimuli: *t*(13) = 0.775, *p* = 0.452; ungrammatical simple v. complex stimuli: *t*(13) = 0.660, *p* = 0.520).

**Table 2:** End-of-sentence judgment task accuracy.

Grammatical stimuli		Mean proportion correct	SE
	Simple	0.93	0.02
	Complex	0.91	0.03
Ungrammatical stimuli			
	Simple	0.96	0.01
	Complex	0.95	0.02

Note: SE = standard error

**Fig 1:**
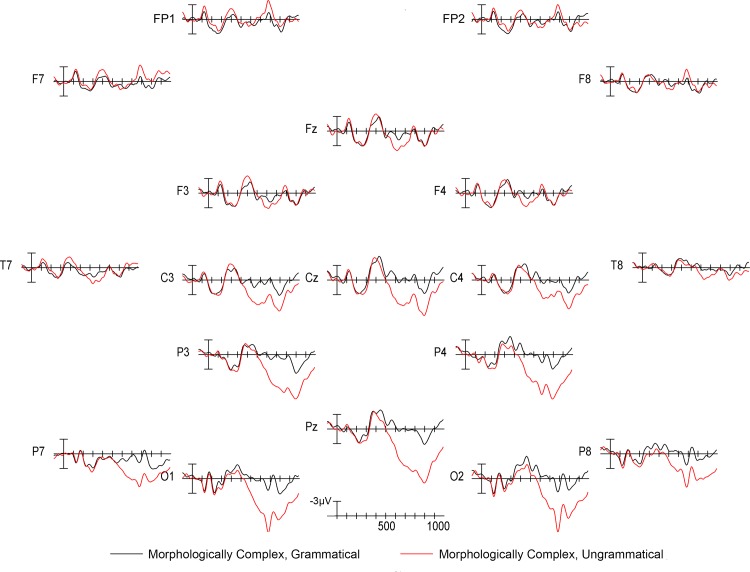
ERP responses to morphologically complex stimuli. Grand mean ERP waveforms for sentences with morphologically complex, grammatical critical words (black line) and sentences with morphologically complex, ungrammatical critical words (red line). Onset of the critical word in the sentence is indicated by the vertical bar. Calibration bar shows 3μVof activity; each tick mark represents 100ms of time. Negative voltage is plotted up.

**Fig 2:**
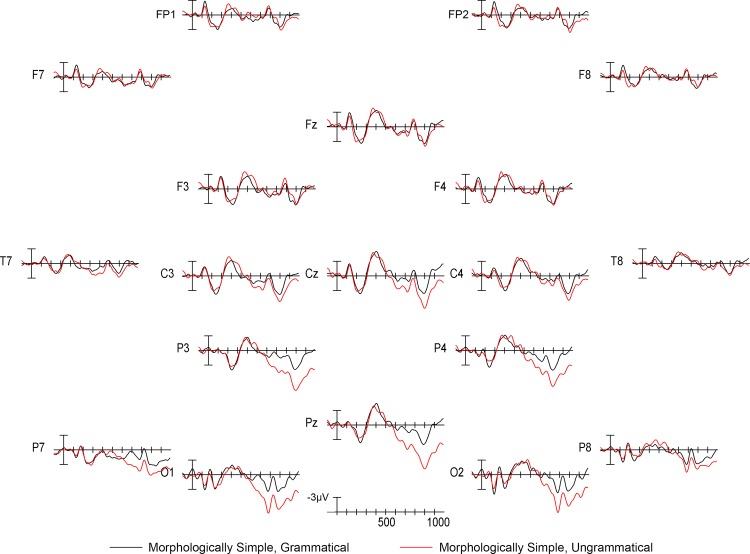
ERP responses to morphologically simple stimuli. Grand mean ERP waveforms for morphologically simple, grammatical critical words (black line) and sentences with morphologically simple, ungrammatical critical words (red line). Onset of the critical word in the sentence is indicated by the vertical bar. Calibration bar shows 3μVof activity; each tick mark represents 100ms of time. Negative voltage is plotted up.

**Fig 3:**
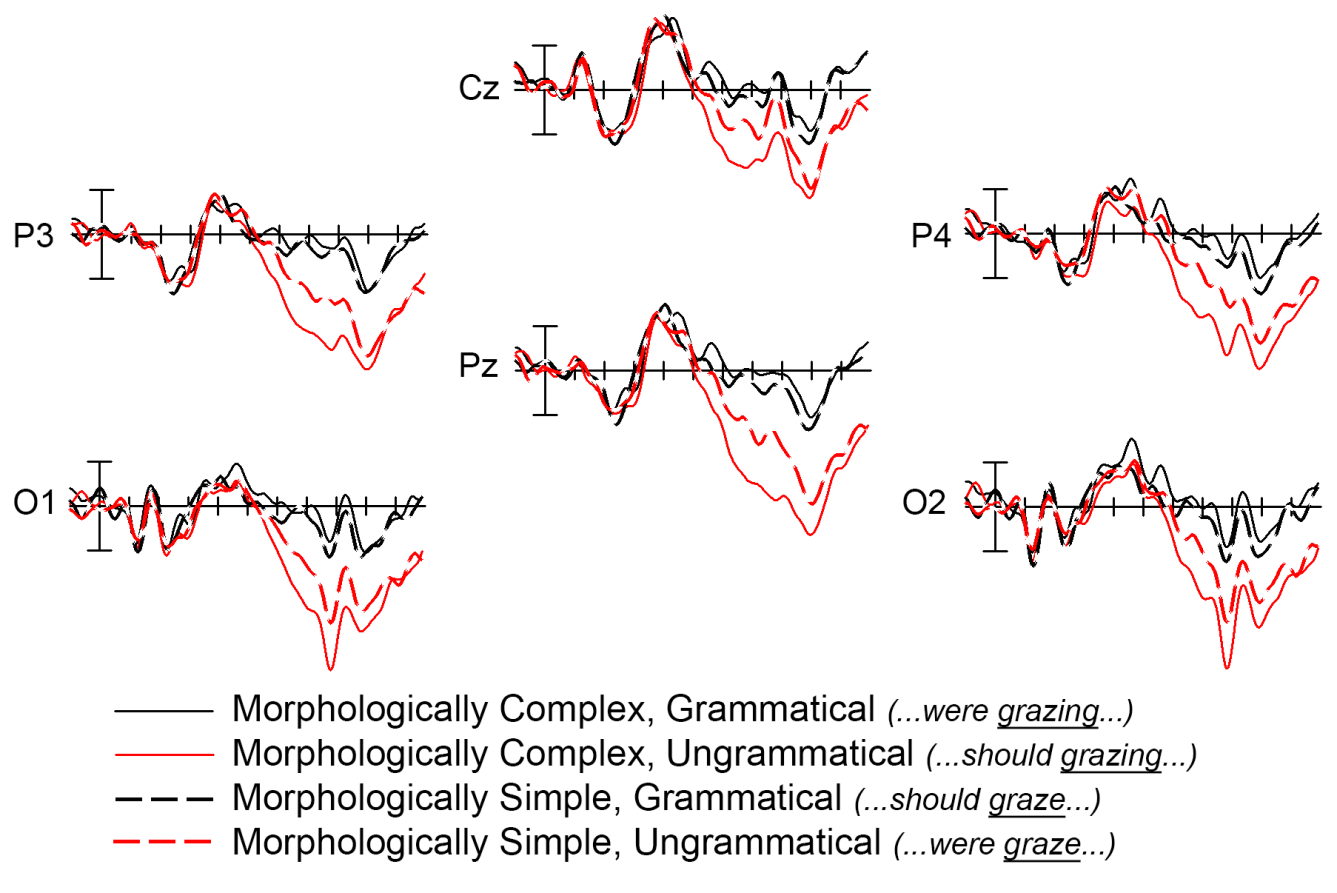
Interaction between grammaticality and morphological complexity. Grand mean ERP waveforms for all four sentence conditions over central parietal and occipital electrodes: grammatical, morphologically simple (black dashed line), grammatical, morphologically complex (black solid line), ungrammatical, morphologically simple (red dashed line), and ungrammatical, morphologically complex (red solid line). Onset of the critical word in the sentence is indicated by the vertical bar. Calibration bar shows 3μVof activity; each tick mark represents 100ms of time. Negative voltage is plotted up.

Visual inspection of the grand mean ERP waveforms showed that relative to grammatical stimuli, ungrammatical stimuli elicited a widely-distributed positivity beginning around 500ms with a posterior maximum (a P600 effect [56]) in both the morphologically complex (Fig 1) and morphologically simple (Fig 2) conditions. The P600 was larger for complex ungrammatical stimuli than for simple ungrammatical stimuli (Fig 3). As can also be seen in Fig 3, there was no difference in the 500–900ms time window between simple and complex grammatical stimuli. Statistical analysis confirmed these observations. In the 500–900ms time window, there was a main effect of grammaticality (midline: *F*(1,13) = 8.278, *p* = 0.013; medial: *F*(1,13) = 6.486, *p* = 0.024) that was strongest over posterior electrodes (grammaticality x electrode interaction: midline: *F*(2,26) = 28.196, *p* < 0.001; medial: *F*(4,52) = 15.606, *p* < 0.001; lateral: *F*(2,26) = 14.038, *p* = 0.001). Also in the 500–900ms time window, there was an interaction between complexity and grammaticality (complexity x grammaticality interaction: midline: *F*(1,13) = 11.467, *p* = 0.005; medial: *F*(1,13) = 14.767, *p* = 0.002; lateral: *F*(1,13) = 5.594, *p* = 0.034), which was again strongest over posterior electrodes (complexity x grammaticality x electrode interaction: midline: *F*(2,26) = 5.818, *p* = 0.015; medial: *F*(4,52) = 7.683, *p* = 0.007; lateral: *F*(2,26) = 13.855, *p* = <0.001).

To further explore the interaction between complexity and grammaticality, we computed the mean activity in all four conditions over a central-posterior region of interest (Cz, P3, Pz, P4, O1, O2) where P600 effects are typically largest [57]. We then performed four paired-samples t-tests and used a Bonferroni correction (alpha level *α* = 0.0125). These t-tests confirmed our initial observations. Relative to grammatical stimuli, ungrammatical stimuli elicited a significant P600, in both the simple and complex conditions (simple grammatical vs. simple ungrammatical: *t*(13) = -3.160, *p* = 0.008; complex grammatical vs. complex ungrammatical: *t*(13) = -5.336, *p* < 0.001) (uncorrected *p*-values are reported). The P600 was significantly larger for complex ungrammatical stimuli than for simple ungrammatical stimuli (simple ungrammatical vs. complex ungrammatical: *t*(13) = -5.564, *p* < 0.001). When comparing the simple and complex grammatical conditions in the P600 time window, one sees that the complex grammatical stimuli were numerically more negative going than the simple grammatical stimuli (note the difference in polarity, relative to the effect of complexity in the ungrammatical sentences). However, this numerical difference was not significant, nor did it approach significance considering the Bonferroni-corrected alpha level (simple grammatical vs. complex grammatical: *t*(13) = 2.013, *p* = 0.065).

Statistical analysis also revealed effects of grammaticality in the 300–500ms time window. Relative to grammatical stimuli, ungrammatical stimuli elicited more negative responses in frontal electrodes and more positive responses in posterior electrodes from 300–500ms (grammaticality x electrode interaction: midline: *F*(2,26) = 7.582, *p* = 0.008; medial: *F*(4,52) = 6.257, *p* = 0.006). There was no effect of hemisphere on this interaction. Independent of electrode site, ungrammatical stimuli produced greater negativities from 300–500ms in the left hemisphere than in the right hemisphere (grammaticality x hemisphere interaction: medial: *F*(1,13) = 6.452, *p* = 0.025; lateral: *F*(1,13) = 6.124, *p* = 0.028). This is similar to some previous reports of left anterior negativity (LAN) effects in response to morphosyntactically anomalous stimuli (see e.g., [9]); however, in the present case left and frontal negativity seemed to be driven by variability in the quality of brain responses (N400 versus P600) to ungrammatical stimuli from individual participants [57,58]. Moreover, LAN effects are highly variable across studies, and recent findings suggest that these left hemisphere negativities can result from component overlap (either within or across individuals) between centrally-distributed N400 effects and right hemisphere-dominant P600 effects [57,58]. Our complexity manipulation did not modulate this negativity, despite visual differences in the magnitude of the negativity (see Figs 1 and 2), likely due to this variability. Instead, it modulated the P600 effect, which, across studies, is a more reliable index of sensitivity to morphosyntactic relations (e.g., [6,8,59]).

## Discussion

This experiment investigated the effects of morphological complexity and grammaticality on the P600 during sentence comprehension. We asked whether manipulations of morphological complexity and stimulus grammaticality would have independent or additive effects on the P600 ERP component, which has been associated with processing both ungrammatical stimuli and grammatical but complex stimuli. Our results showed a strong interaction between these two factors, with no discernible unique effects of morphological complexity on the P600. Specifically, we found that whereas both morphologically simple and complex ungrammatical words in sentences elicited a P600 (as expected), the P600 was larger for morphologically complex ungrammatical words than morphologically simple ungrammatical words. There was no reliable difference in brain responses between the morphologically simple and complex grammatical conditions. These results have both methodological and theoretical implications.

Methodologically, these results indicate that care must be taken when comparing the effects of grammaticality when the stimuli being compared vary in morphological complexity. This is especially relevant when comparing the magnitude of the P600 across different experiments, and should be taken into account when stimuli are designed. As advocated for by Steinhauer & Drury [17], using a balanced stimulus design, in which simple and complex forms of the critical verb are each used in grammatical and ungrammatical conditions, overcomes the confound of morphological complexity (example from [17]: Grammatical: 1) *“The boys* *play* *a game*.*”*, 2) *“The boy* *plays* *a game*.*”*; Ungrammatical: 3) *“The boys* *plays* *a game”*, 4) *“The boy* *play* *a game*.*”*). While resolving the morphological complexity confound, this type of stimulus design may lead researchers to miss subtle differences in processing associated with different levels of complexity on the target. For example, Marcinek and colleagues [60] recently reported subtle differences in ERP responses across complexity conditions in sentences like those described above. Specifically they report that in comparisons of grammatical simple and ungrammatical complex subject-verb agreement manipulations (*The boys* walk/*walks *…*), ERP results showed a LAN followed by a P600, but when comparing grammatical complex to ungrammatical simple agreement manipulations (*The boy* walks/*walk…), there was no apparent LAN, and the P600 had an earlier onset.

These cautions are not simply limited to cases of morphological complexity, but extend to other linguistic features as well. Importantly, in some cases, averaging across sub-conditions within balanced designs could lead to spurious theoretical conclusions when sub-conditions elicit qualitatively different brain responses. For example, Marcinek and colleagues [60] further report that asymmetric word category violations in a balanced design elicit qualitatively different brain responses: nouns substituted for verbs (*… to* enjoy/*meal *…*) elicit N400 effects, whereas verbs substituted for nouns (…*the* meal/*enjoy *…*) elicit P600 effects. Nieuwland, Martin and Carrieras [61] show that animacy alternations, which require differential object marking in Spanish, elicit qualitatively different brain responses: animate object nouns preceded by determiners unmarked for animacy (…al/*el *obispo* *…*, “the bishop”) elicited N400 effects, whereas inanimate object nouns preceded by animate-marked determiners (…el/*al *suelo* *…*, “the floor”) elicited P600 effects. In either of these cases, simply averaging across levels within the balanced design would have resulted in either spurious biphasic effects or null effects, depending on the level of spatiotemporal overlap of the N400 and P600 effects. Thus, as the present data additionally suggest, some caution is warranted when interpreting data averaged across conditions within balanced designs. In some cases this averaging would obscure subtle differences in processing (as in the present data), or more gravely result in averaged ERP waveforms not reflective of participants’ processing in the sub-conditions of the balanced design (see [15,57,62] for further cautions about averaging across individual participants within conditions who show qualitatively different ERP responses to linguistic violations). This is not to say the balanced designs should be abandoned altogether, simply that researchers must consider the consequences that the averaging procedure may have on their final interpretation of the data.

At a theoretical level, the present data clearly show that the processing of verb ungrammaticality is not a unitary phenomenon. Instead, the P600 elicited by ungrammatical verbs is sensitive to the morphological complexity of the target word. One possible theoretical account for this pattern is provided by the prediction/memory search account of morphosyntactic processing outlined in the Introduction. On this account, predicted features, even in complex contexts, do not trigger processing difficulties. In the two grammatical contexts of the present experiment, there was no statistically reliable difference in P600 amplitude and no other evidence of processing difficulty in the ERP record. This is despite differences in the stimuli at the level of morphological complexity, as well as more superficial levels (e.g., word length). However, when bottom-up encountered input requires retrieval of previously encountered input (e.g., in the case of unexpected or ungrammatical morphosyntactic features, or in the processing of verb-argument relations in filler-gap configurations, or other complex dependencies), P600 amplitude should index salience of the anomaly and also difficulty in establishing a licit dependency. Among the factors implicated in this process are the strength to which comprehenders commit to the initial prediction and the level of cue overlap between the critical word and target. All else being equal, more difficulty in processing (indexed by larger P600 amplitudes) should occur when initial predictions were stronger (and thus more difficult to overcome) and when salient retrieval cues of the probe do not match with any potential targets (see [8,42,51,52] for other recent similar results in this vein).

In the present study P600 amplitude was significantly larger for ungrammatical stimuli in the morphologically complex condition (…should * *grazing* *…*) than in the morphologically simple condition (…were * *graze* *…*). We can account for this by appealing to both of the above-mentioned constraints: predictability and cue overlap. It is well-established that comprehenders predict upcoming information, and that the constraints imposed by the linguistic context guide not only comprehenders’ expectations of upcoming material, but also the strength to which they commit to those expectations. This has been most thoroughly explored in the realm of sentential constraint on semantic and lexical predictions. Several studies have now shown that comprehenders actively use semantic context to predict both upcoming lexical items, as well as semantic, syntactic, and phononlogical features of those items [34,35,63–69]. Importantly, the level of lexical and semantic constraint that a sentence context provides is approximately linearly associated with N400 amplitude, and even varies as a function of the number of alternate completions even across sentences with similar constraints (as measured by cloze probability) [63]. The role of prediction is made even clearer in work by Brothers, Swaab and Traxler [70]. They show that accuracy in prediction shows earlier effects on brain responses than contextual effects, measured by cloze probability for discourse-final words. Prediction accuracy for final words modulated the N250, whereas contextual constraint effects were not present until the N400 time window.

Effects of prediction strength have also been demonstrated in the realm of grammatical processing, particularly when evaluating the role of statistical probabilities on the processing of verbal complements. Several studies have shown effects of verb-complement co-occurrence bias both behaviorally [71–73] and using ERPs [7]. Specifically, Osterhout, Holcomb and Swinney [7] demonstrated that the statistical probability of a given verb occurring with a sentential versus NP complement modulated P600 amplitudes. Osterhout and colleagues showed graded P600 amplitudes to disambiguating verbs in complement clauses based on probability that main clause verb will occur with complement clauses: the largest P600s were found following verbs that never occur with sentential complements, intermediate P600s were found following verbs that occur most frequently with NP complements (but still occur occasionally with sentential complements), and no P600 effects were found following verbs lacking a particular complement bias. This demonstrates that linguistic experience can shape processing behavior at the syntactic level, where P600s elicited by violations of expectations show amplitudes in inverse proportion to the strength of those expectations.

In the present study we can see the effects of predictability by considering the morphosyntactic constraints imposed by the pre-critical auxiliary verbs in our stimuli. English modal verbs (e.g., *can*, *could*, *shall*, *should*, *will*, *would*) occur primarily in frames where they subcategorize for verbs in their bare stem form, whereas finite forms of the verb *be* (e.g., *is*, *are*, *were*) are far less restrictive in the types of complements they occur with. They can co-occur with participial verbal complements (as in our grammatical stimuli), as well as with predicative adjectives or nouns, with prepositional phrases, etc. To obtain a course-grained approximation of the level of constraint imposed by the two types of verbs, we queried the Corpus of Contemporary American English [74] to estimate the proportion of tokens where finite forms of *be* (*am*, *is*, *are*, *was*, *were*) are followed within two words by a present participle (-*ing* form) as well as the proportion of tokens where modal auxiliary verbs (*can*, *could*, *shall*, *should*, *will*, *would*, *must*, *might*, *may*) are followed within two words by a verb in its base form. The proportion for finite forms of *be* was 0.135; the proportion for modals was 0.346. That is, encountering a finite form of *be* predicts that a verbal present participle will be encountered within two words 13.5% of the time, whereas encountering a modal predicts that a base verb form will be encountered within two words 34.6% of the time. The local subcategorization constraint imposed by modals is thus approximately three times that imposed by finite forms of *be*. This suggests that, as demonstrated by Osterhout, Holcomb and Swinney, participants’ prior experience with English co-occurrence probabilities shape their expectations about upcoming material and the strength to which they commit to those expectations. Violation of these stronger predictions thus results in enhanced P600 effects.

The second determinant of P600 amplitude within the prediction/working memory search account implicates cue overlap between retrieval probes and potential targets. Within a content-addressable memory retrieval architecture (e.g., [44,48]), in order to be available as a retrieval target, the cues available on an item must match a subset of those available in working memory. Work within the sentence comprehension literature has shown that both syntactic and semantic features can serve as retrieval cues when establishing morphosyntactic and other verb-argument dependencies [8,49,50]. Prior ERP work in this vein has shown that P600 amplitude is related to potential cue-overlap when processing subject-verb agreement: P600 amplitudes are larger in ungrammatical contexts when there are no potential targets for cues than when a potential target is held in memory, even when it is not a syntactically licensed agreement controller [8,75]. In the context of the current experiment, a search of working memory is triggered upon encountering ungrammatical verbs: when encountering syntactically unexpected verb forms, comprehenders search working memory to seek out an appropriate licensor of those features (i.e., to potentially recover a mis-comprehension). In our stimuli, verbs in the simple condition are underspecified for features, relative to verbs in the complex condition. Participial verbs contain specifications for aspectual features (e.g., telicity, boundedness), which must be syntactically and/or semantically licensed [76]. Based on these featureal specifications, participial verbs must “seek out” particular targets that license their features; as participles mismatch in licensing features with preceding modal verb contexts (…should * *grazing* *…*), large ungrammaticality effects results. In contrast, base verbal forms occur in many syntactic contexts (finite, nonfinite, singular, plural, etc) and thus are relatively free in their syntactic distribution. Being underspecified for features, base verb forms thus neither match nor mismatch with features in preceding context, resulting in reduced effects of ungrammaticality (cf. [76,77] for formal approaches to underspecification).

The results from the present study cannot determine unambiguously whether strength of prediction or feature mismatch may have contributed independently or in an interactive fashion in shaping the results here. Note also that the prediction/memory search account is only one of a number of possible explanations for the observed interaction in our data. However, our present data align nicely with a growing body of research implicating both prediction and retrieval as key determinants of sentence processing success and difficulty [8,36–38,42,44,46,51,52,70,78,79]. Future research will be needed to more clearly establish the exact cognitive locus of these effects.

More importantly, our results clearly demonstrate that ungrammaticality of verb forms is not a unitary processing phenomenon. Instead, neural responses to ungrammatical verbs clearly varied as a function of the morphological cues and complexity of the critical word, whereas brain responses to grammatical verbs did not show a similar sensitivity. A further limitation of this experiment is that the morphologically complex words (e.g., *eating*), are not only more complex but also longer than the morphologically simple words (e.g., *eat*). This could result in ungrammaticalities in the complex condition being simply visually more salient than in the simple condition. However, there is sufficient reason to believe that length alone cannot account for the effects we see in our data, and in particular, the enhanced P600 wave in the complex ungrammatical condition compared to the simple ungrammatical condition. First, if length had any unique effect on ERPs in our data, it should be visible in a comparison of the two grammatical conditions (e.g., *should* *graze* versus *were* *grazing*). In our data, there were no significant differences between these two conditions in the P600 time window, suggesting that length alone cannot explain the effects we saw in the ungrammatical conditions. Moreover, to the extent that length had any effect on the data, it was numerically opposite with respect to the grammaticality condition. That is, the complex (and thus longer) word in the grammatical condition was numerically (but non-significantly) more negative-going, but in the ungrammatical condition it was very reliably more positive-going. If there were systematic effects of length on the P600 effect, they should have shown the same polarity with respect to the grammaticality manipulation; instead, we found opposite polarity effects, indicating that length alone cannot explain the significant P600 amplitude differences in the two ungrammatical conditions.

In summary, this experiment has shown that whereas there is no unique effect of morphological complexity on the P600, overt morphological cues (e.g., -ing) elicit a larger P600 when stimuli are ungrammatical, compared to when the overt cues are not present. These results have important methodological considerations for ERP research, and also provide more information about what types of cognitive processes the P600 may be indexing.

## Supporting Information

S1 Table All experimental and filler sentences. All four versions of each experimental sentence, as well as the 60 filler sentences used in every list.
(XLSX)

S2 Table ERP mean amplitudes and participant characteristics. Mean ERP amplitudes for each participant in the 300–500ms and 500–900ms time windows in all four sentence conditions at all electrodes, as well as each participant’s gender and age.
(XLSX)
